# Study on numerical simulation and mechanical properties of anchor cable with C-shaped tube subjected to shearing

**DOI:** 10.1038/s41598-024-58085-9

**Published:** 2024-03-28

**Authors:** Renliang Shan, Shengchao Xiao, Yongzhen Li, Xinpeng Zhao, Tianyu Han, Shupeng Zhang

**Affiliations:** China University of Mining and Technology (Beijing), Beijing, 100080 China

**Keywords:** Anchor cable, Anchor cable with C-shaped tube, Deformation and failure characteristics, Numerical simulation, Stress development law, Civil engineering, Structural materials

## Abstract

To examine the disparity in deformation behavior and mechanical qualities between anchor cables with C-shaped tubes and regular anchor cables under shear conditions. The double-sided shear tests of free-section anchor cables and anchor cables with C-shaped tubes were conducted utilizing the indoor large-scale double-shear test equipment with varying pretension loads. The indoor double-shear tests indicate that the inclusion of the C-shaped tube alters the stress distribution of the anchor cables inside the anchor cables with C-shaped tubes and mitigates the impact of stress concentration. Moreover, it facilitates the transition of the anchor cable's failure mode from a mix of tensile and shear breaking to mainly tensile breakage. In addition, ABAQUS finite element analysis software was used to establish a double shear test model of the anchor cable with C-shaped tube to accurately simulate the interaction and stress distribution among the anchor cable, C-shaped tube, and concrete block in the double shear test. The findings of the simulation results reveal that the numerical model can adequately depict the evolution of the stress distribution in the prestressed anchored structure and the damage of the concrete block with increasing shear displacement. The relational equation for the yield state of the anchor cable with C-shaped tube under combined tensile and shear loads is found by integrating the experimental and simulation data, the static beam theory, and the concept of minimal potential energy.

## Introduction

In the context of underground excavation projects, such as mining and tunnel engineering, the layout of passageways is typically situated within the rock mass. The presence of joints and fissures within the rock mass reduces its strength, leading to the potential for relative movement of fractured rocks. For instance, the movement of vertical joints can trigger a phenomenon known as delamination, while the movement along parallel joints may result in shear sliding. Rock bolt and anchor cable support structures are effective methods for controlling rock deformation, enhancing the stability and load-bearing capacity of fractured rock mass structures, and ensuring the overall stability of geotechnical engineering structures. Particularly in recent years, mining operations have increasingly ventured into deeper strata, where the prevalence of fractured rock masses and strata sliding along joint planes subjects anchor bolt and cable support constructs to heightened lateral shear forces. This elevates their susceptibility to failure and significantly magnifies the risk of severe engineering catastrophes. The study of shear strength in anchor rod and anchor cable structures and the deformation and failure mechanisms of support components under joint shearing is of significant importance for advancing the development of support technologies^1–4^.

The mechanical properties of shear along support structures and joint planes have garnered significant attention from numerous scholars, leading to extensive research on this subject. Aziz et al.^5–7^ studied the shear mechanical properties and deformation failure characteristics of various anchor rods and cables under full-length anchorage conditions using an improved novel single and double shear test apparatus. Craig et al.^8^ conducted bilateral shear tests on 28 mm hollow strand "TG" type anchors, revealing the load transfer mechanisms prior to structural failure and identifying that the failure of the anchors was due to axial loads reaching their ultimate capacity. Li et al.^9,10^ applied the superstatic beam theory to establish an analytical model of shear strength and shear displacement of anchor cable joints with full-length anchorage and investigated the effects of parameters such as preload force, joint friction angle, concrete strength, anchor cable installation angle, and type of anchors on the shear strength of the joint plane. Rasekh et al.^11^ explored the effect of preload, anchorage length, number of strands, and type of anchors on the total shear strength of the support structure under frictionless conditions at the nodal plane. The above research results on the full-length anchorage of the support component shear mechanical properties and deformation law of the detailed study for the future to further deepen the study of the mechanical properties of anchor rods and anchor cables to provide a worthy reference to the experimental means and research methodology. However, most of its research objects are full-length anchored anchor rods and anchor cable structures, and there are few studies on the shear mechanical properties of the free section of the anchor cable.

Due to the intricate compositional nature of anchor cable systems and their high-strength mechanical properties, conducting indoor shear tests poses particular challenges. Consequently, numerical simulation research methods have become favored among many scholars for their study. Li et al.^12^ employed the FLAC3D software to conduct numerical modeling of fully grouted rock bolts, investigating the effects of rock mass strength, bolt inclination, and bolt diameter on the shearing behavior. The results obtained were in good agreement with those from laboratory tests. Sun et al.^13^ employed the FALC3D software for secondary development to analyze rock bolts' bearing capacity, yielding numerically, and fracture under tension-shear coupling effects. Proposed a constitutive model for the yielding and fracture of rock bolts and established a novel numerical model for rock bolt breakage under combined tension-shear loading. Nie et al.^14^ established an anchorage model through the DDA framework, which predicts the bonding strength of bonded-type anchor bolts and the axial load. The model shows a reasonable degree of fit. Aziz et al.^15^ conducted a study on the simulation of the mechanical behavior of prestressed anchors under different installation angles by secondary development of the 3DEC and UDEC software. Tahmasebinia et al.^16,17^ employed ABAQUS finite element analysis software to establish a dual shear test model for anchors, facilitating the investigation of the shear failure characteristics of anchors under impact load conditions. Numerical simulations often employ simplified models for anchor cable structure investigations. These models reduce the complex anchor cable structure to a single rod element, disregarding the interactive relationship between individual strands composing the anchor cable structure. Additionally, the focus of these studies is predominantly on the anchorage section of the anchors.

**Figure 1:**
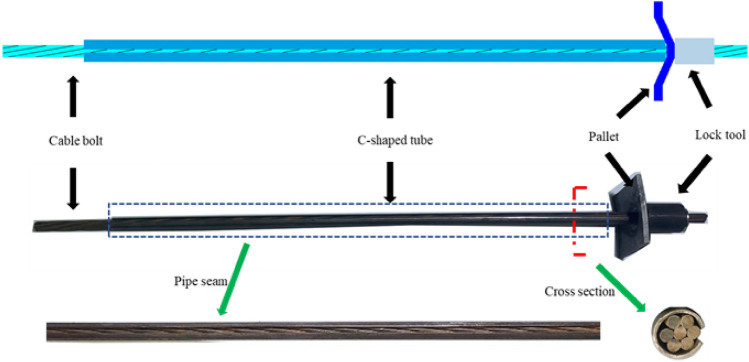
Schematic illustration of the anchor cable with C-shaped tube.

The majority of current study results focus on the anchorage section of typical anchor cable constructions, with just a few studies examining the shear mechanical features of their free section. In addition, research on improving the total shear strength of joint plane also focuses on methods such as augmenting the pretension force of anchor cables and the quantity of support components. Few studies primarily focus on the structure of support components, aiming to enhance their shear strength and improve the overall shear performance of joint surfaces. Therefore, Shan Renliang et al.^18–22^ took the supporting member as the primary research body and invented a new type of tube and cable combination structure (The full name is anchor cable with C-shaped tube, ACC for short. In the following sections, ACC will be used to refer to the anchor cable with C-shaped tube). To enhance the shear strength of the free section of the anchor cable, as shown in Fig. 1. It is a specialized support system designed to withstand the transverse shearing forces exerted by the surrounding rock mass in underground coal mining engineering, tunnels, and galleries. Its constituent structures mainly include an anchor cable, a C-shaped tube, a locking device, and a shim.

In contrast, research in numerical modeling has focused on simplifying the anchor cable structure to a single bolt, disregarding the interaction among the numerous strands. The development of internal structural stresses during shear tests needs to be explored in sufficient depth. In this study, leveraging the unique characteristics of the ACC structure, a comparative analysis is conducted on the shear deformation characteristics of both ordinary anchor cables and the ACC structure. Drawing on the outcomes of experimental studies, ABAQUS finite element analysis software was utilized to simulate the ACC structure. This simulation aims to explore the evolution law of its deformation process and to inform the development of underground engineering support technologies.

## Double-shear test of support structures

To investigate the differences in deformation processes between ACC structures and conventional anchors under shear loads within rock masses, as well as the effects of varying pre-tensioning forces on both structures, double-sided shear tests were conducted on the anchorage systems composed of the two types of constructions, under pre-tensioning forces of 100 kN, 200 kN, and 300 kN respectively.

### Test preparation

**Figure 2:**
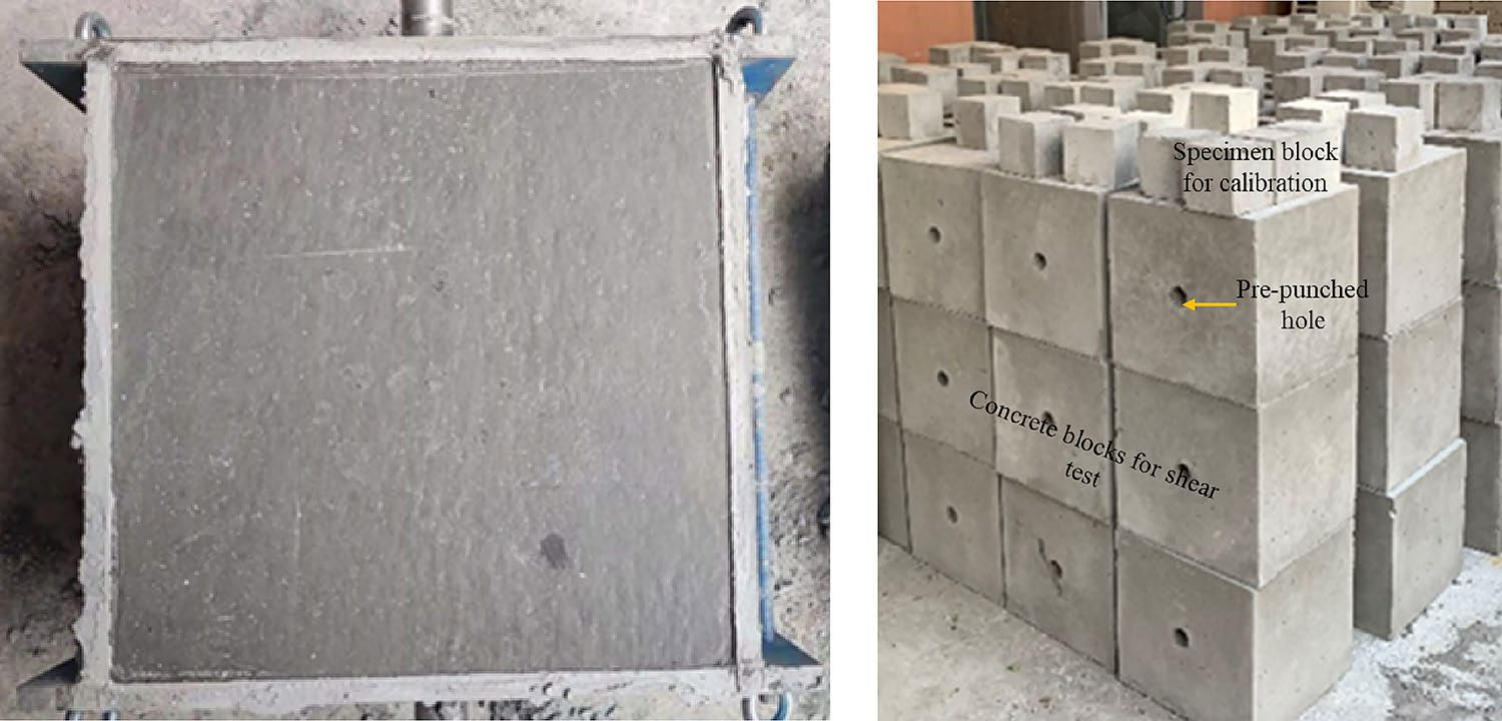
Fabrication of concrete blocks.

The anchoring system comprises a prestressed anchor cable/ACC structure and concrete blocks. The prestressed anchor cable is constructed from seven strands of twisted steel wires. In the experimental setup, the ACC structure's anchor cables share identical model specifications and mechanical properties with conventional anchor cables. The prestressed anchor cable/ACC structure is installed at the central position within the concrete blocks. The concrete blocks are manufactured through a uniform mixing procedure that combines water, cement, coarse aggregate with a particle size of less than 10mm, and fine sand in a proportion of 1:2:4:4, respectively. The production process is shown in Fig. 2. Upon fabrication, the specimens are subjected to curing at ambient temperature for 28 days prior to testing. The concrete block has a uniaxial compressive strength of 47 MPa, and it is composed of three segments: left, middle, and right, each with dimensions of 300 × 300 × 300 mm. When casting concrete blocks, holes are incorporated to make installing prestressed anchor cables and ACC structures easier.

**Figure 3:**
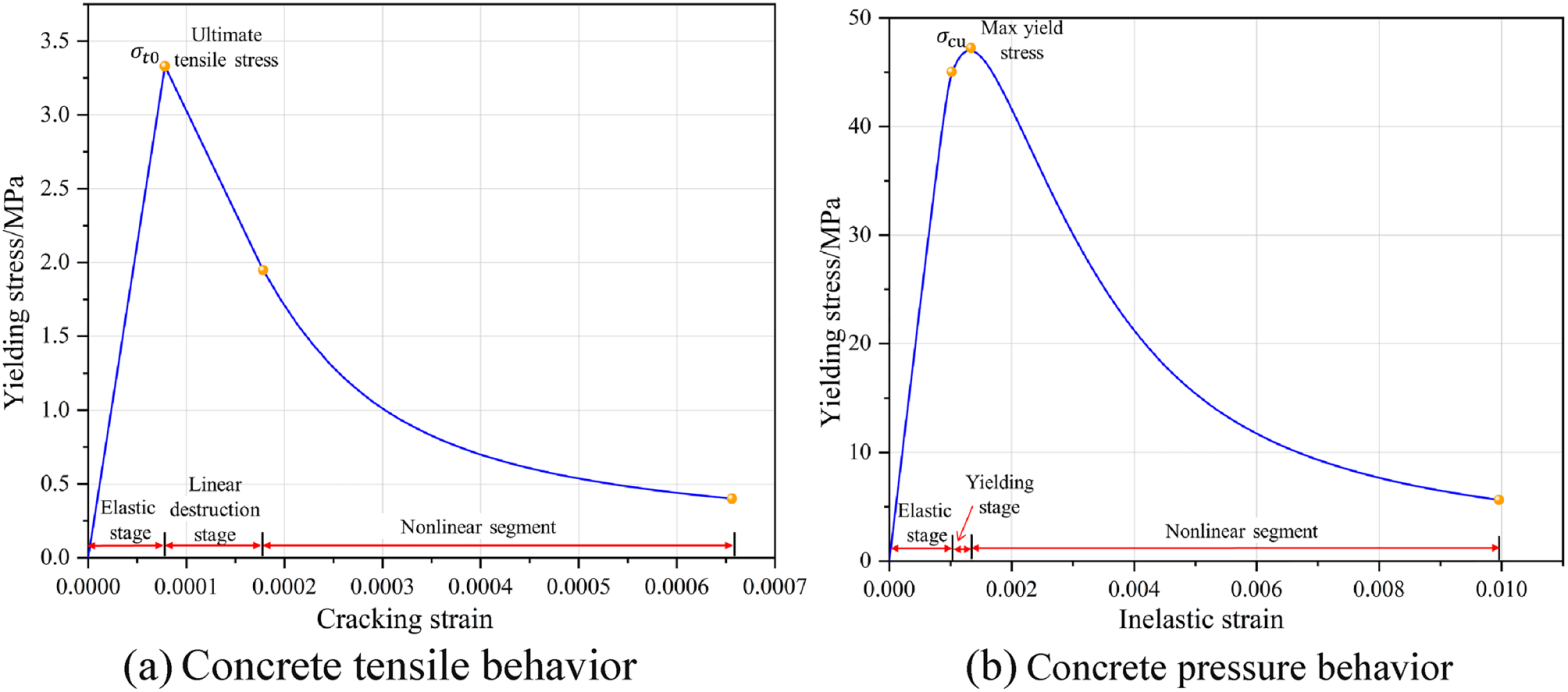
Concrete stress-strain curve.

**Figure 4:**
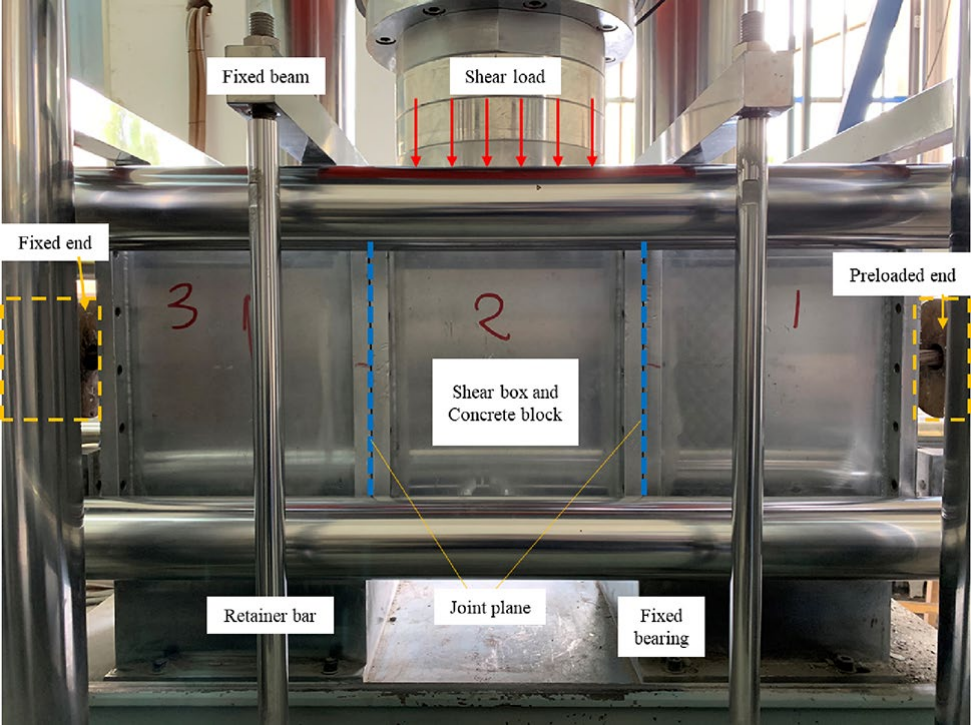
Physical diagram of the test system.

The physico-mechanical parameters of the concrete block are presented in Table 1. According to laboratory testing, the uniaxial compressive strength of the concrete specimen was measured as 47 MPa. The stress-strain curve of the concrete, as obtained from laboratory experiments, is shown in Fig. 3. The tensile testing of concrete encompasses three distinct phases: the linear elasticity stage, the linear damage stage and the nonlinear damage stage. The compression test curve of concrete is usually divided into linear elastic stage, yield stage and nonlinear failure stage. The tension tests on the anchor cables and the shear tests on the anchorage structures were conducted using a cable-rod tension-shear test system designed by China University of Mining and Technology (Beijing). During the double-sided shear test, the anchoring structure needs to be put into the shear box made of high-strength steel to be fixed to ensure the test's safety. The experimental setup is illustrated in Fig. 4. The shear load was applied during the experiment by controlling the shear displacement rate. The loading rate for shear displacement was established at 2 mm/min. The shear load was measured by sensors mounted on the vertical loading end and transmitted to the computer control system.

**Table 1:** Parameters of concrete blocks.

Density, *ρ*(kg/m^3^)	Elastic modulus, *E*/GPa	Poisson ratio, ${\varvec{\mu}}$	Friction angle, $\boldsymbol{\varphi }$/°	Cohesion, *c*/MPa	Compressive strength, $\sigma$/MPa
2400	32.5	0.2	38	1.0	47

### Anchor cable tensile test

**Figure 5:**
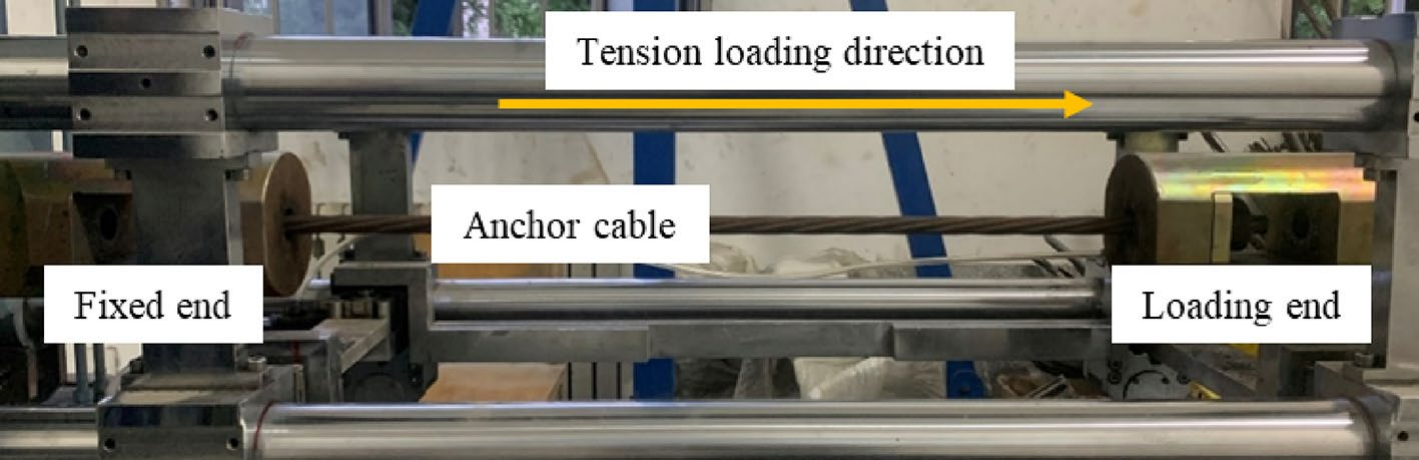
Anchor cable tensile test setup.

**Figure 6:**
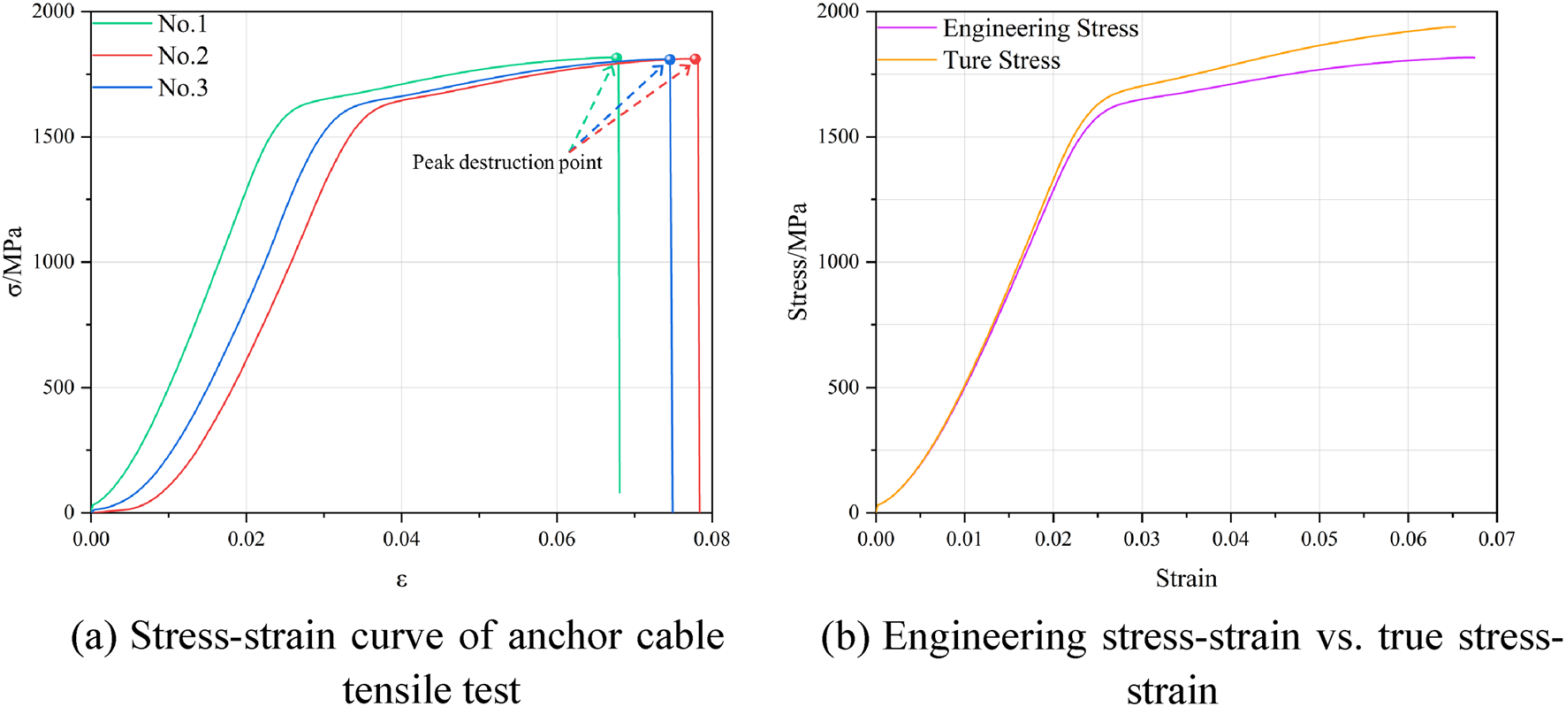
Tensile stress-strain curve of anchor cable.

Before conducting the double-shear tests on anchorage structures, it is necessary to perform tensile tests on the anchor cable systems. The relevant physico-mechanical parameters were determined. Due to the inherent characteristics of ACC structures, the C-shaped tubes only bear shear forces during the shearing process; the axial forces they experience are negligible. Consequently, subjecting the internal anchor cables to tensile testing is only necessary. Therefore, the tensile test is also conducted using the tubular strand rod tension-shear test system, as illustrated in Fig. 5. During the anchor cable tensile test, both ends are secured by locking devices, with the right end being the loading side. The effective tensile length of the uniaxially stretched anchor cable specimen is 1085 mm. Tensile experiments were performed on three anchor cables, and the corresponding stress-strain curves are presented in Fig. 6a. Owing to the limitations of the engineering stress-strain curve obtained in the laboratory, its value does not accurately reflect the changes in cross-sectional area due to the material's plastic damage. Furthermore, to enhance the calibration of the numerical model used in simulations, conversion of the engineering stress-strain curve to the true stress-strain curve is imperative. Equations (1) and (2) below are employed to transform the engineering values of the material into true values, and these transformed values are juxtaposed with the true stress-strain curves displayed in Fig. 6b.

1 $$\varepsilon = ln\left( {1 + \varepsilon_{nom} } \right),$$

2 $$\sigma = \sigma_{nom} (1 + \varepsilon_{nom} ),$$

where $\varepsilon$ is the true strain value; ${\varepsilon }_{nom}$ is the engineering strain value; $\sigma$ is the true stress value; ${\sigma }_{nom}$ is the engineering stress value.

The physico-mechanical parameters of the anchor cable determined through laboratory testing are presented in Table 2. The C-shaped tube, fabricated from Q345b strip steel, has physico-mechanical parameters that can be sourced directly from the ABAQUS software's material library. Table 3 displays the geometric and physico-mechanical characteristics of the C-shaped tube.

**Table 2:** Anchor cable parameters.

Density, *ρ*(kg/m^3^)	Elastic modulus, *E*/GPa	Breaking stress, $\sigma$/MPa	Length, *L*/mm	Cross-sectional area, *S*/mm^2^	Diameter, *D*/mm
7800	202.4	1920	1400	285	21.6

**Table 3:** C-shaped tube parameters.

Density, *ρ*(kg/m^3^)	Elastic modulus, *E*/GPa	Slit width, *b*/mm	Length, *L*/mm	Outer diameter, *D*_*1*_/mm	Inside diameter, *D*_*2*_/mm
7850	210	8	1000	285	21.6

### Double-sided shear test results and analysis

**Figure 7:**
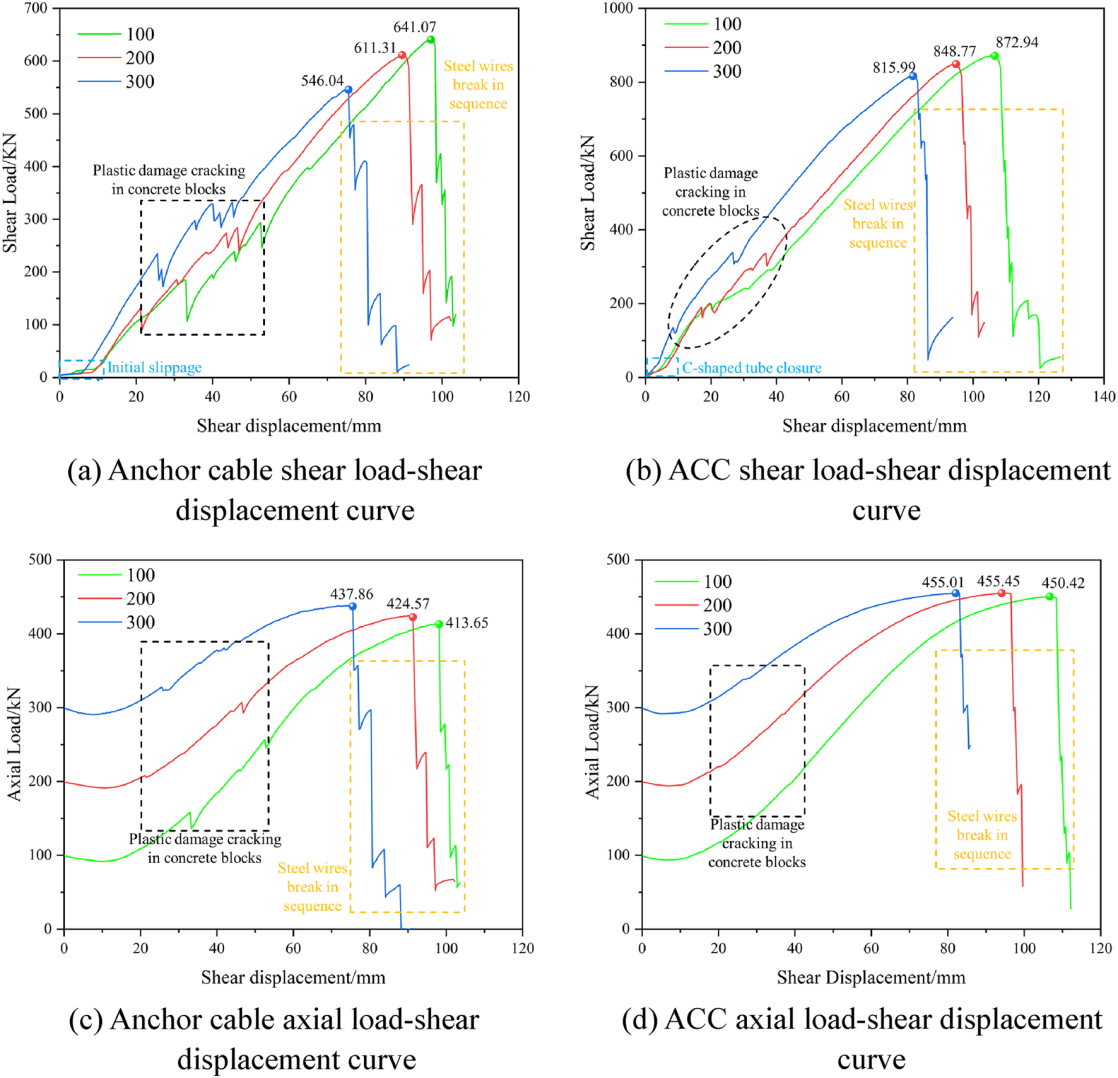
Shear-displacement and axial force-displacement curves of anchor cable and ACC structure.

The test result curve is shown in Fig. 7. The shear load was administered at the onset of the double-sided shear test and maintained until the termination of the assessment upon the failure of all supporting elements, which endured for approximately 50 minutes. During testing, the anchor cable fractured on a total of 7 occasions. Introducing a gap between the concrete specimens during testing negates any potential impact of friction on the test results. Test data were digitized to generate shear load-versus-shear displacement curves for both ordinary anchor cables and ACC structures under varying preloads, along with the axial load-versus-shear displacement curves.

**Figure 8:**
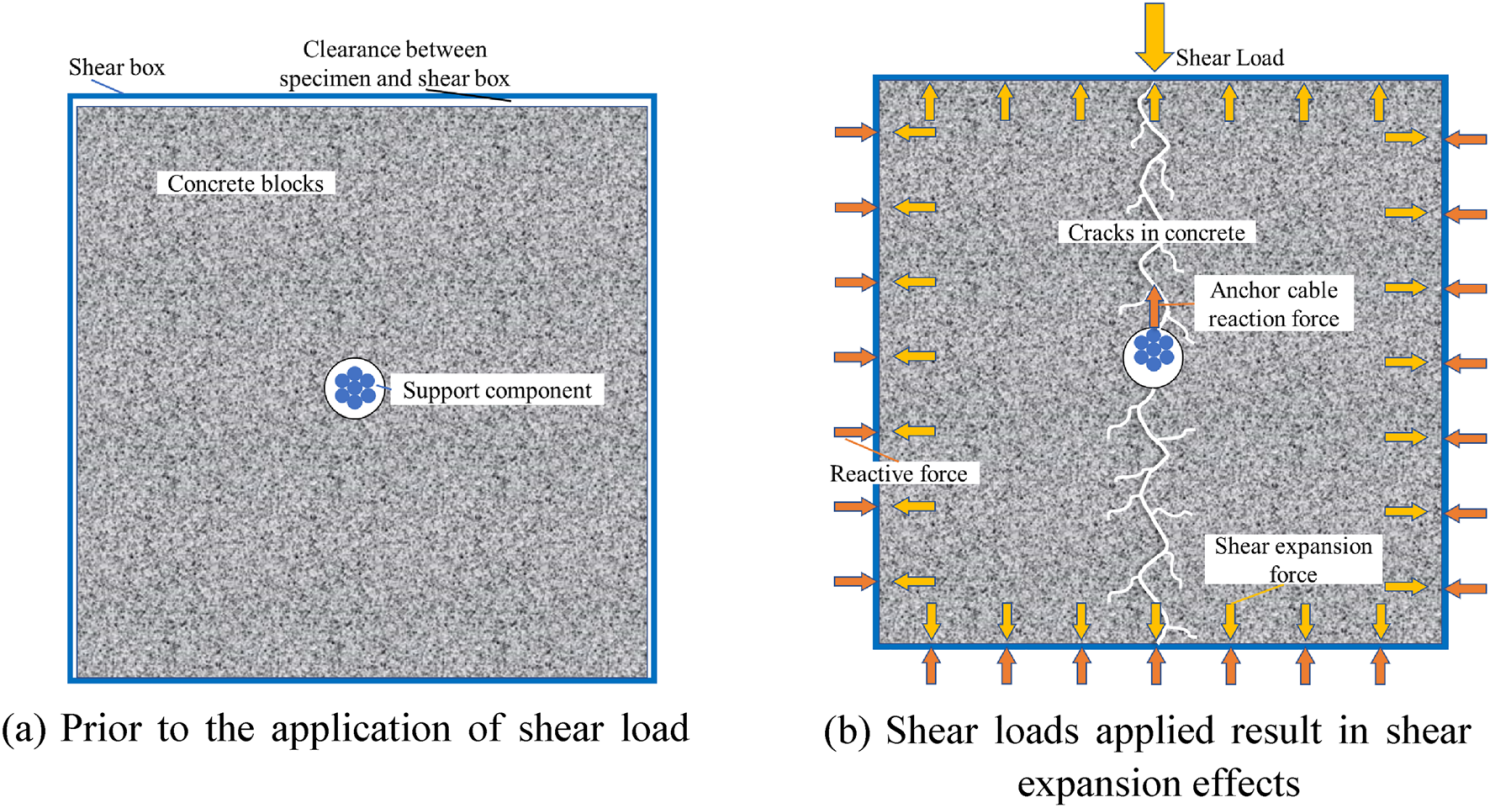
Schematic of shear expansion effect.

In the double-sided shear test of two support component types, a pronounced fluctuation was observed in the curve section where the shear load ranged from 100 to 350 kN. In this section, the concrete block initiated significant plastic deformation and exhibited signs of cracking. Post this phase, attributable to the shear expansion effect depicted in Fig. 8 and the presence of the shear box, a restraining effect is imposed on the concrete block. This restraint mitigates the progression of extensive cracking and spalling. Consequently, the subsequent curve's fluctuation is attenuated or potentially eliminated. Compared to standard anchor cables, the ACC structure exhibits a more subdued fluctuation within the 100 to 300 kN curve segment. This is tentatively attributed to the C-shaped tube enhancing the contact interface between the ACC assembly and the concrete. Simultaneously, the smooth exterior of the C-shaped tube distributes the force across the contact zone more evenly and diminishes the stress concentration. Fluctuations in the curve’s descending segment occur as the strands sequentially fail upon reaching their peak load-bearing threshold.

**Figure 9:**
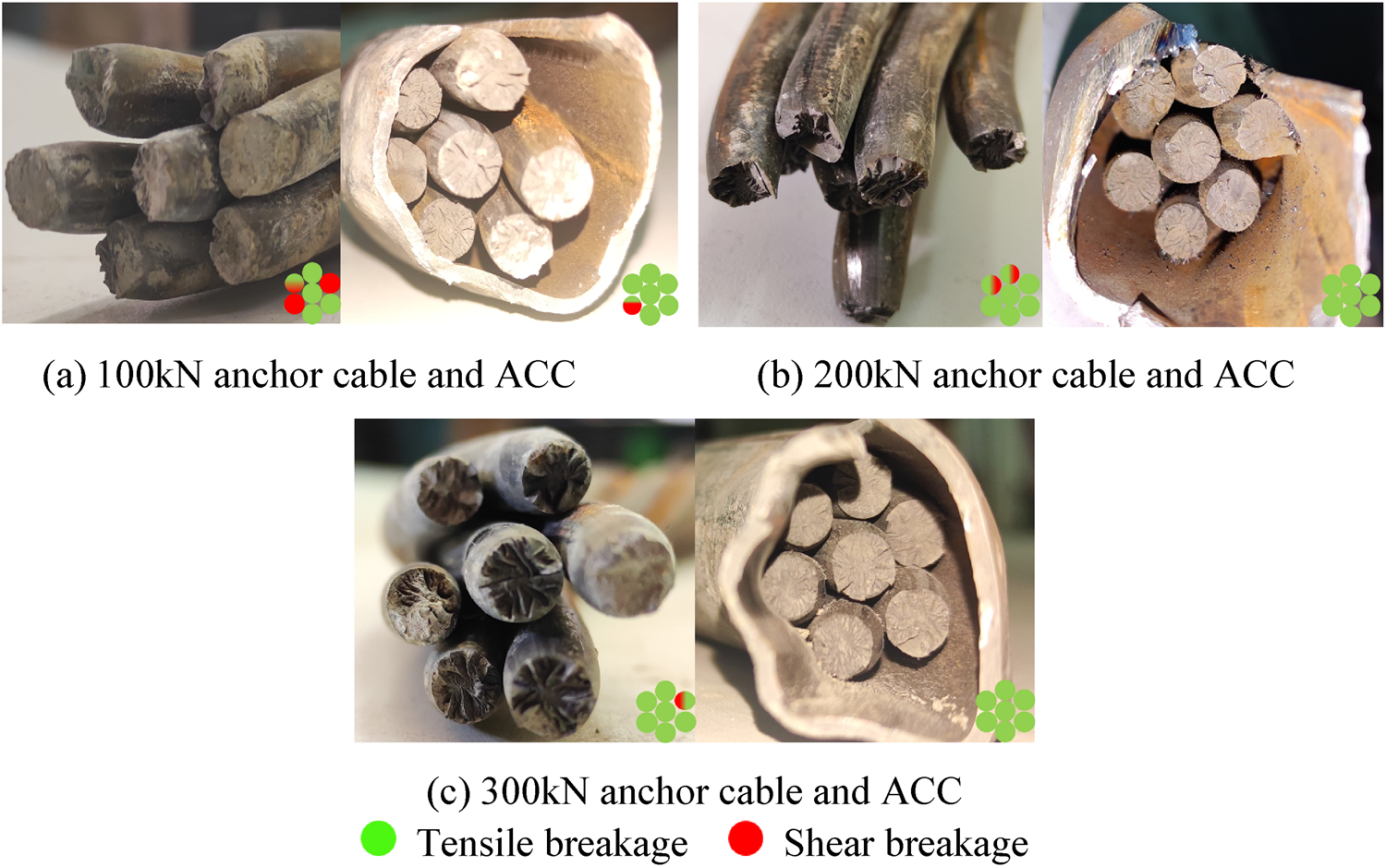
Anchor cable and ACC structure shear test results.

The axial load-shear displacement curves indicate that the ACC structure possesses a superior tensile load capacity compared to the conventional anchor cable. The ultimate tensile loads of the ACC structure exhibit consistency across three different preload conditions. This also suggests that integrating the C-shaped tube confers increased shear strength upon the ACC structure while concurrently augmenting the tensile strength of the internal anchor cables. The most important point is to improve the stress state of the anchor cable structure and make the anchor cable structure under shear loading conditions from the combination of tensile and shear breakage form to the main form of tensile breakage conversion. This conclusion can be corroborated by the result of Fig. 9 from shear tests conducted on both plain anchor cables and ACC structures under three different prestressing conditions.

### ACC shear strength exploration

**Figure 10:**
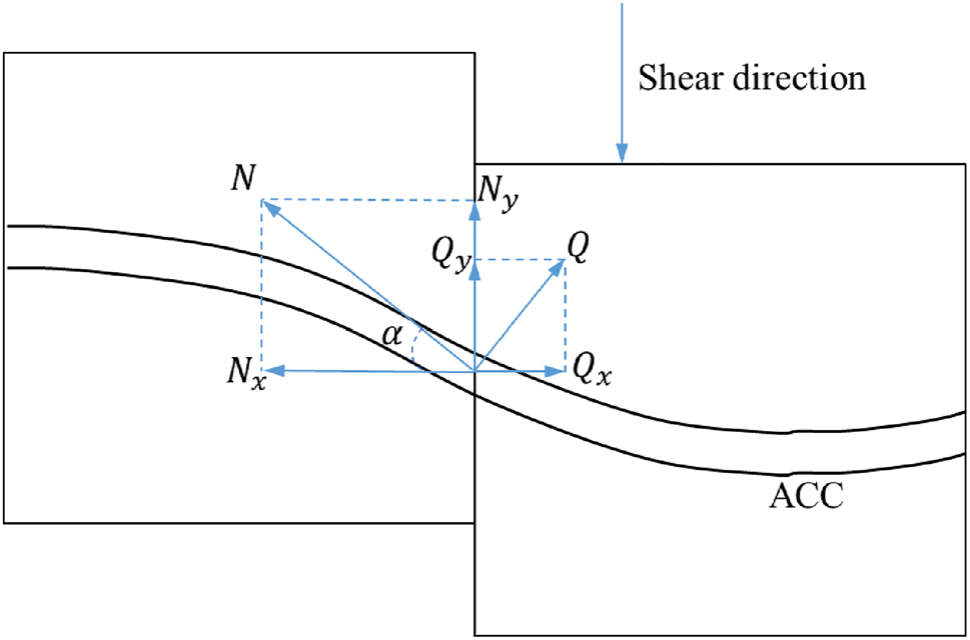
Schematic diagram of structure-rock interaction.

In the standard shear test, the interaction between structure and rock mass is shown in Fig. 10. The composite shear strength $\tau$ at the interface comprises three distinct elements^23^. Shear strength ${\tau }_{1}$ is provided by the nature of the rock joint plane itself in the normal direction, shear strength ${\tau }_{2}$ provided by the axial force of the support structure, and the shear strength provided by the shear force ${\tau }_{3}$, the relationship can be expressed as:

3 $$\tau = \tau_{1} + \tau_{2} + \tau_{3} ,$$

As a result of the spaces between the structural surfaces in this experiment, eliminating the influence of the inherent characteristics of the rock joint plane and the friction between joints on the shear strength of the structural surface. Therefore, the shear strength at the nodal plane of the ACC structure:

4 $$\tau = \tau_{2} + \tau_{3} + \tau_{c} ,$$

5 $$\tau_{2} = \frac{{N_{o} \sin \alpha }}{{A_{B} }},$$

6 $$\tau_{3} = \frac{{Q_{o} \cos \alpha }}{{A_{B} }},$$

7 $$\tau_{c} = \frac{{F_{s} - N_{o} \sin \alpha - Q_{o} \cos \alpha }}{{A_{c} }},$$

${N}_{o}$ is the tensile force applied to the anchor cable at breakage, $kN$; ${Q}_{o}$ is the shear force applied to the anchor cable at breakage, $kN$; ${A}_{B}$ is the cross-sectional area of the anchor cable, ${mm}^{2}$; ${A}_{c}$ is the cross-sectional area of the C-shaped tube, ${mm}^{2}$; ${F}_{s}$ is the shear load applied when the ACC structure is broken,$kN$; ${\tau }_{c}$ is the shear strength provided by the C-shaped tube.

Analysis of the experimental data for the shear and axial load curves of the ACC structure reveals a positive correlation between axial load and ${\tau }_{2}$. Meanwhile, the relationship between ${\tau }_{c}$ and ${\tau }_{3}$ and their links to the individual components remains elusive based on current testing and warrants further investigation via numerical simulation.

## Numerical simulation analysis

### Numerical model creation

The ABAQUS 3D numerical simulation software was utilized to model the progression from preload application through shear load application to the ultimate failure of the ACC structure and concrete block. To more effectively address the contact issues among individual structures and enhance convergence, the "explicit dynamics" algorithm within the software was employed to ascertain the mechanical behaviors of the ACC structure under shear loading. The model was established using a 1:1 scale, matching the dimensions of the laboratory test specimen. The geometric parameters for the concrete block have been set at 300 mm × 300 mm × 300 mm.

The mesh elements used for the concrete, anchor cables, and C-shaped pipes in the numerical model are all C3D8R type. The C3D8R element is an octahedral element with excellent deformability and stress analysis capabilities. It also possesses good convergence properties and stable computational performance, allowing for more accurate simulation of the deformation of complex structures and providing reliable analysis results. For the contact between different structures in the finite element model, the "general contact" formulation was chosen. To prevent model penetration, the "hard contact" option was selected for the normal behavior. The "penalty" attribute was chosen for the tangential behavior, and different friction coefficients were assigned. The data defined in the damage model was obtained based on the stress-strain curve and the built-in ABAQUS user manual.

**Figure 11:**
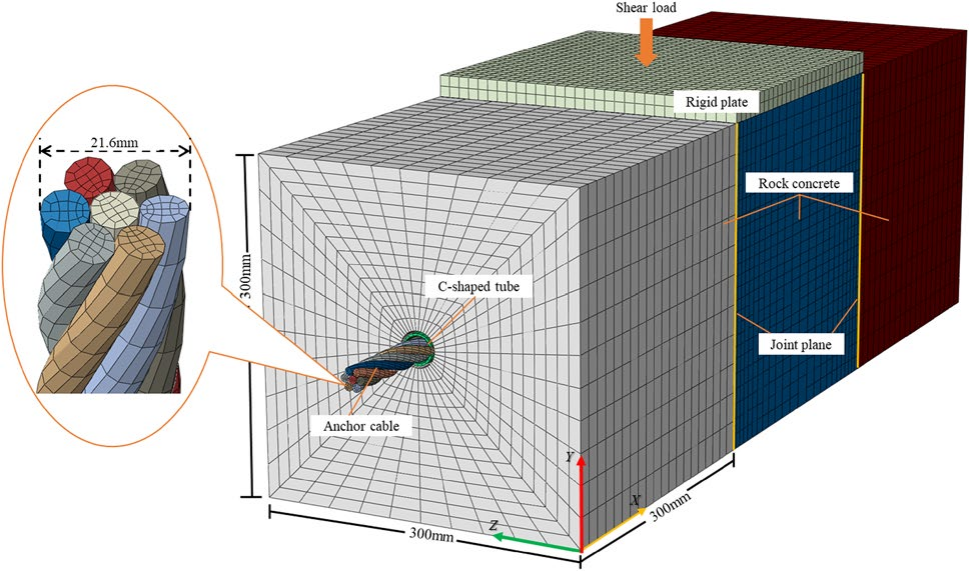
Numerical model.

The anchor cable element adopts 20% of the diameter of the steel strand structure as the element size. The C-shaped tube elements adopt 10% of its actual length as the element size, and the concrete block elements adopt 4% of the actual length of the specimen as the element size^16^. Lastly, the software's internal grid quality evaluation tool is used to calibrate it. The final meshing of the model is shown in Fig. 11.

Boundary condition settings for the model: The left and right concrete block models featured fully constrained supports on the faces at Y = 0, Y = 300, Z = 0, and Z = 300; The central concrete block has displacement constraints in the X and Z directions on the same four Y and Z planes mentioned previously. Displacement loading in the Y-direction simulated the loading mode of the test laboratory, with a displacement loading rate set at 2 mm/min, corresponding to the laboratory's standard rate.

### Numerical model verification of ACC structure

**Figure 12:**
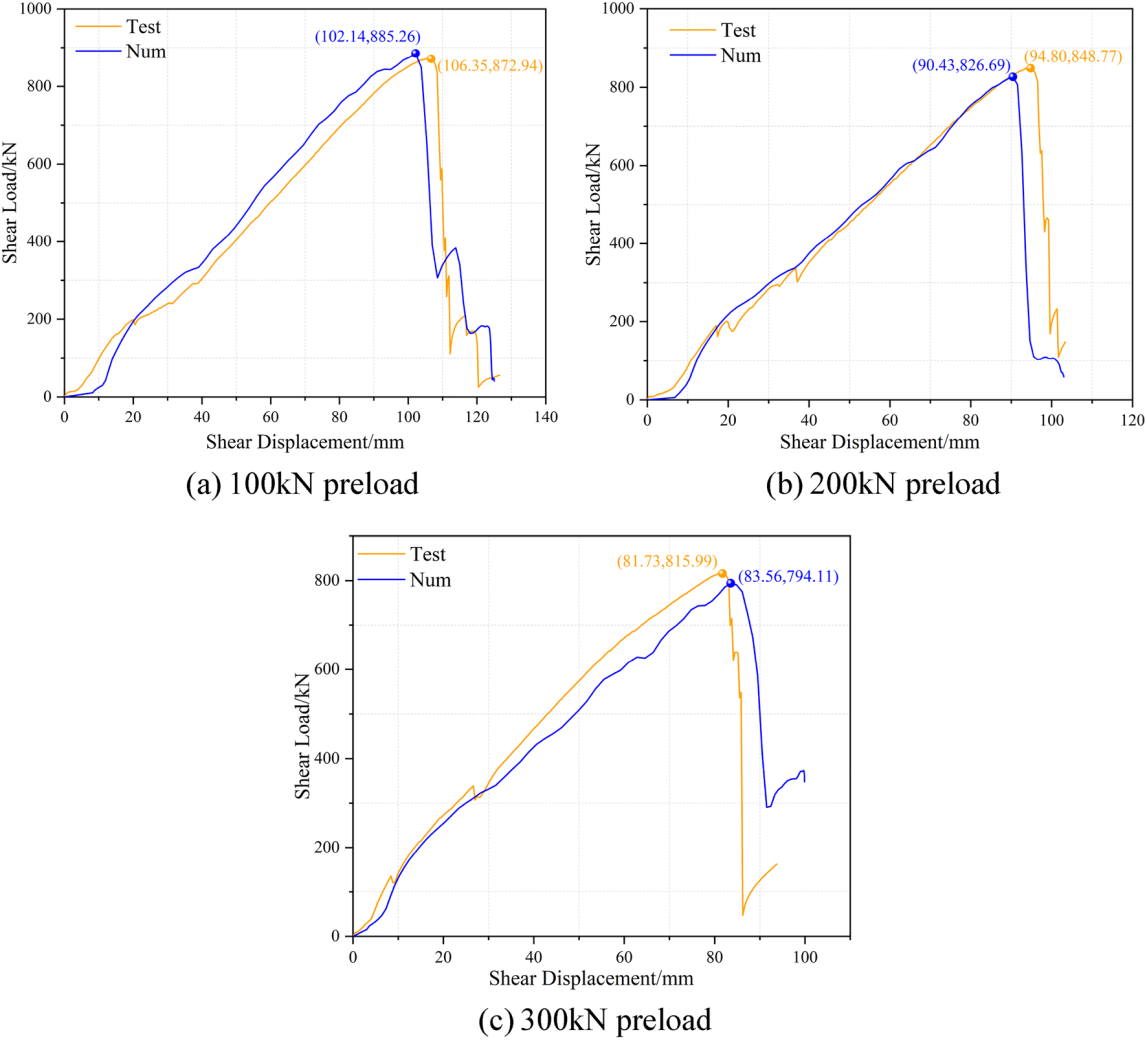
Comparison curve between numerical simulation results and laboratory test data.

The numerical simulations conducted in this segment of the research utilized the model established in the prior modeling section, ensuring property consistency with previous scenarios. In the case of changing only the magnitude of the prestressing force, comparative analysis of the load changes observed during the shear test between the numerical model of the ACC structure and the laboratory-based in-situ model. A comparison of the shear load-shear displacement curves derived from numerical simulation and those from the laboratory tests is presented in Fig. 12.

Overall, the numerical simulation results have achieved a good fit in terms of peak shear load and shear displacement. Concerning the progression of the curve, the numerical simulation outcomes succinctly capture critical phases in the shear deformation process of the ACC structure, exhibiting a progression that is primarily in agreement with the indoor test curve. The numerical simulation curve clearly depicts the phenomenon that the internal anchor cables within the ACC structure undergo sequential failure after reaching peak shear strength. This custom is particularly pronounced in the simulation with a 100kN preload. In the numerical simulation results for the 300 kN prestress, the latter half exhibits a trajectory marginally below that of the indoor experimental curve. The primary reason for this phenomenon is the damage inflicted on the concrete block by the ACC structural shear, with a portion of the shear energy being dissipated throughout the concrete block's deformation and subsequent damage.

### Structural deformation analysis of ACC

**Figure 13:**
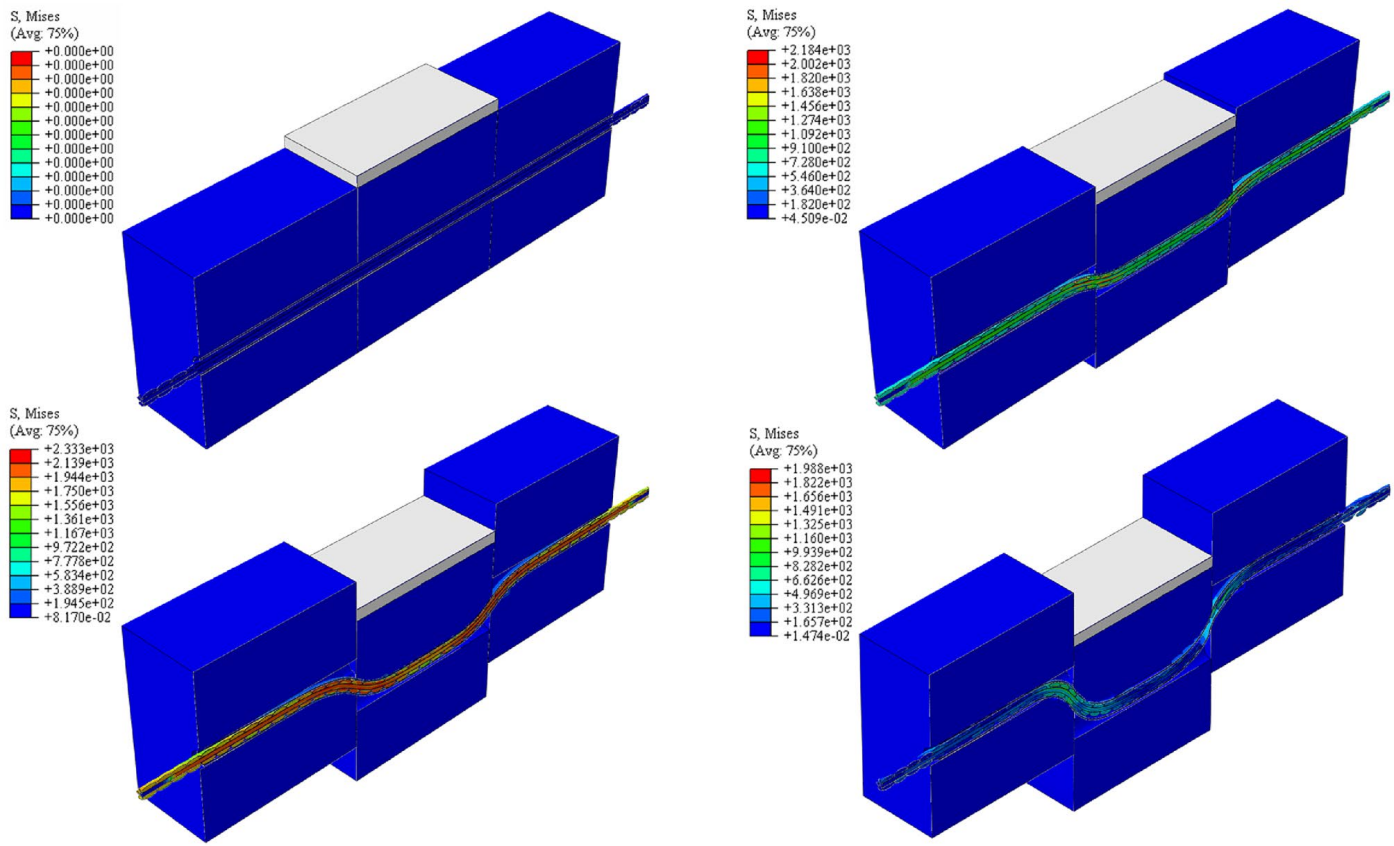
Numerical model deformation process.

Figure 13 presents a schematic diagram depicting the numerical simulation for the double-sided shear test on the ACC structure subjected to a 200 kN prestressing force, spanning from the initial application of the shear load to the point of structural failure. The legend on the left side of Fig. 13 represents the distribution and evolution of Mises stress in the ACC structure during the double shear test.

The ACC structure developed a symmetrical distribution of plastic regions at the junction with the structural face of the concrete block throughout the loading process. The central concrete block initially contacts the ACC structure and transfers the shear load through the C-tube to the internal anchor structure, subsequently distributing it to the concrete blocks on both the left and right sides. As shear displacement increases, the concrete structure exhibits significant plastic deformation and damage, the C-tube structure begins to close gradually, the anchor cable structure undergoes deformation, and the ACC structure throughout the shear region assumes a "seagull" configuration. At any joint plane, the ACC structure exhibits an "S" curve shape at the joint plane, and the stresses within the overall structure are symmetrically distributed along the joint planes on both sides, respectively.

The loads and deformations experienced by the ACC structure throughout the test are divided into the following stages: In the first stage, the internal anchor cables are tensioned under prestressing, and the axial force of these cables gradually increases until it reaches the expected load; In the second stage, as shear displacement increases incrementally, the C-shaped tube progressively constricts, and the ACC initiates the formation of a plastic hinge structure on both sides of the structural surface, anchor cable axial force initially tends to decrease. In the third stage, the shear strength at the plastic hinge of the ACC structure diminishes progressively until it is nullified, and the axial load on the anchor cable commences its increase, however, it does not achieve the ultimate tensile capacity; In the fourth stage, the ACC structure starts to damage, the C-shaped tube breaks and the internal anchors start to fail one after the other until the axial load is reduced to zero. The deformation behaviors observed during the ACC structural shear tests under prestress levels of 100 kN and 300 kN are analogous.

### Deformation and failure analysis of concrete blocks

**Figure 14:**
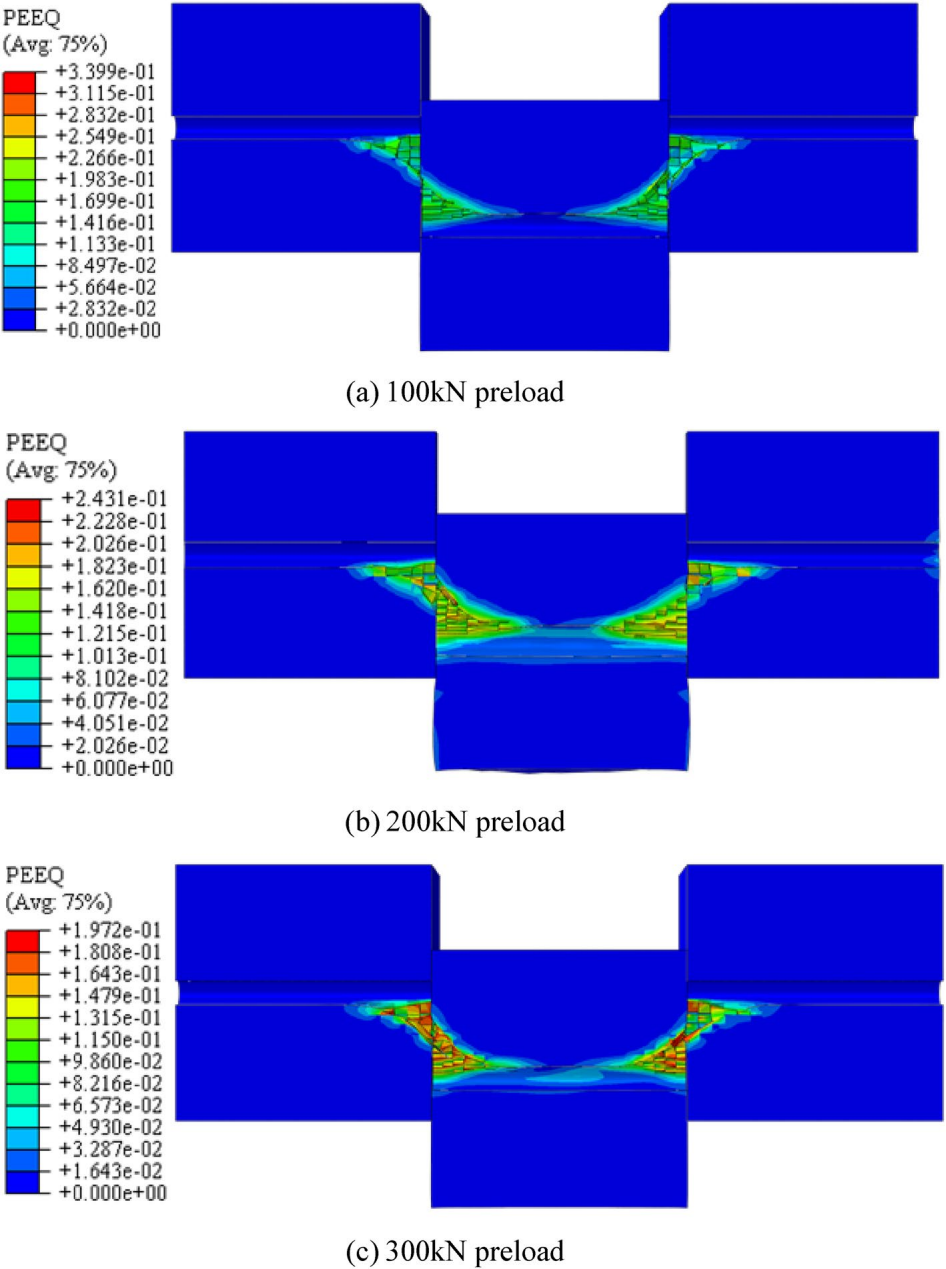
Distribution of plastic zone of the concrete block under three preload forces.

The degree of yielding damage experienced by the rock mass during shearing varies with each of the three distinct preload forces. Concrete was used to simulate rock to represent the perimeter rock damage process in the ACC structure accurately during the double shear test. Furthermore, the concrete, plastic damage model is applied within the numerical simulation to depict the plastic deformation damage of the concrete block. Figure 14 depicts the yield zone of the concrete block at the interface of the structural face and the ACC structure when subjected to the three preload forces. The legend on the left side of Fig. 14 represents the distribution of equivalent plastic strain regions in the concrete block.

The extent of damage to the rock mass correlates with the material strength of the rock, among other factors. For the purposes of this numerical simulation, the concrete material’s yield strength has been configured at 47 MPa. Therefore, from Fig. 13, it can be found that the distribution of the yield region of numerically simulated concrete blocks is roughly similar for the three preload conditions. The degree of yielding decreases with increasing preload force. The yield degree gradually decreases as the preload increases. This phenomenon can be understood by recognizing that, as the preload increases, the ACC structure requires less shear energy to fracture, resulting in reduced shear displacement. Consequently, the concrete block in the entire shear test process exhibits a gradually reduced degree of yield and amount of deformation within the plastic-yielding region.

**Figure 15:**
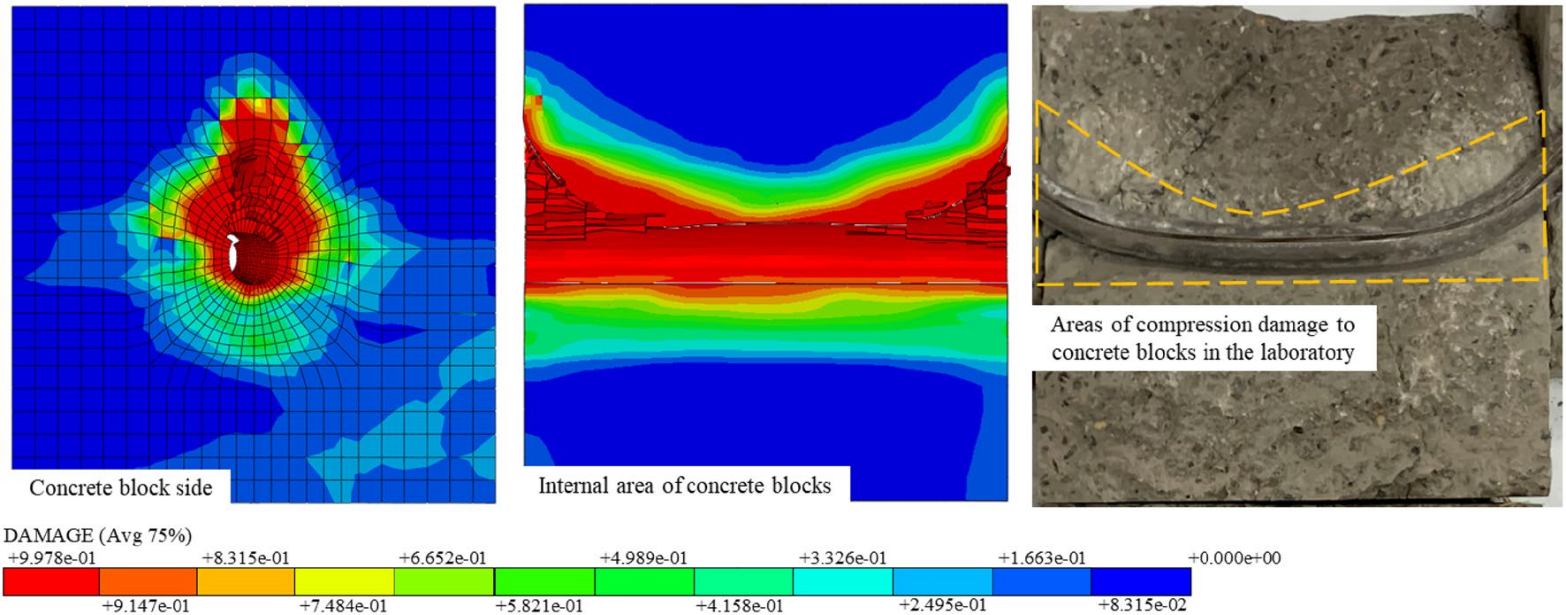
Comparison between numerical simulation and test in the damaged area of the concrete block under compression damage.

Meanwhile, the deformation and damage patterns of concrete blocks obtained from the simulation correspond closely to those observed in the test chamber. The area of compression damage in the concrete block during the shear process of the ACC structure under a 200kN preload is depicted in Fig. 15. The legend below, Fig. 15, represents the distribution of the compressive failure regions in the concrete. The distribution area predominantly occupies the upper part of the concrete block, with a pattern resembling the yield region. The profile adequately illustrates the evolution of the compression damage area within the concrete as the ACC structure deforms during the shearing process, ultimately manifesting as a "U" shape. The lateral view shows the conical outward expansion of the area affected by compression damage.

### Stress distribution of ACC structure

**Figure 16:**
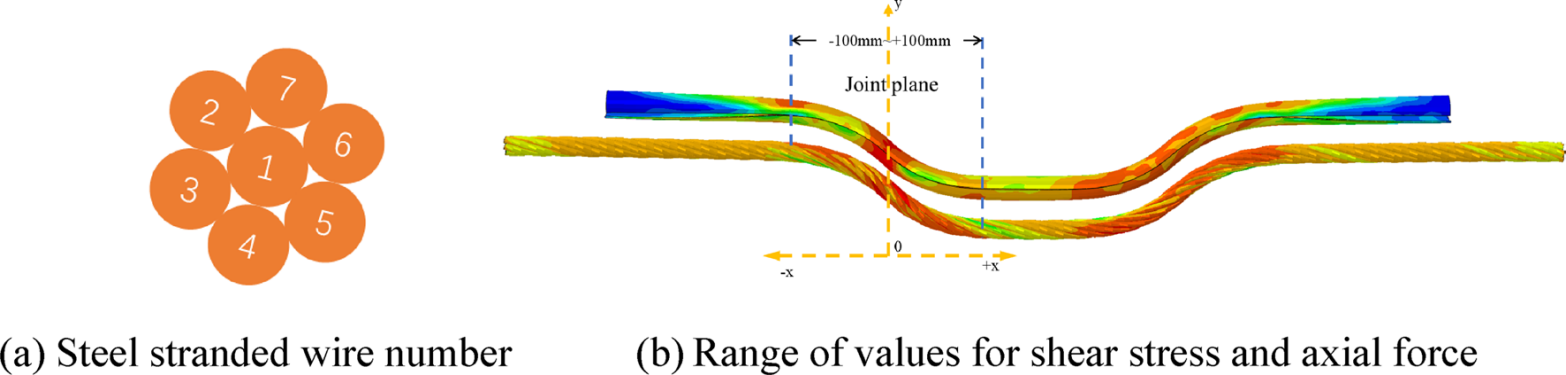
Schematic diagram of strand numbering and stress extraction area.

**Figure 17:**
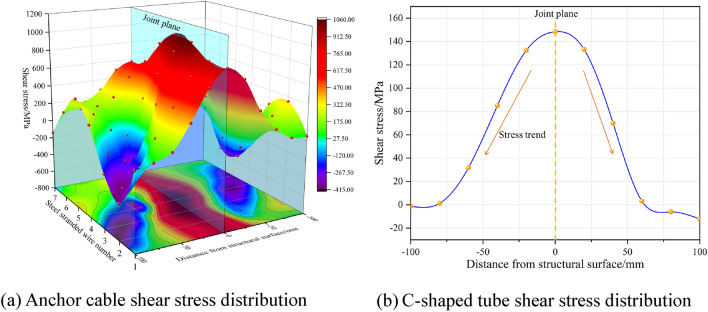
Distribution of shear stresses in ACC structural components.

Due to the specific nature of the ACC structure and the complex nature of laboratory tests, it is currently infeasible to monitor the internal stress changes throughout the ACC structure during the double shear test process. In particular, the distribution of shear and axial stresses during structural deformation and damage requires using numerical simulation methods to explore and analyze thoroughly. In the numerical simulation, the strands comprising the anchor cables within the ACC structure are assigned numbers, as depicted in Fig. 16a. The axial and shear stresses of the seven strands and the C-shaped tube, measured at distances of 0, ± 20, ± 40, ±60, ±80, and ±100 mm from the joint plane before the breakage of the ACC structure, as shown in Fig. 16b, were extracted and defined as representative stresses. Figure 17 shows the shear stress distribution from the joint plane at different locations.

As illustrated in Fig. 17, the peak shear stresses of the strand and the C-tube occur within a range of ± 20 mm from the structural surface. With increasing distance from the joint plane, the shear stresses in both the strand and the C-tube diminish markedly and exhibit a symmetrical distribution centered around the joint plane. Figure 17a also reveals that the fluctuation in shear stress distribution for the central No. 1 strand is minimal, whereas the outer strands No. 2~7 experience significant non-uniformity and pronounced fluctuations in shear stress due to torsional effects.

**Figure 18:**
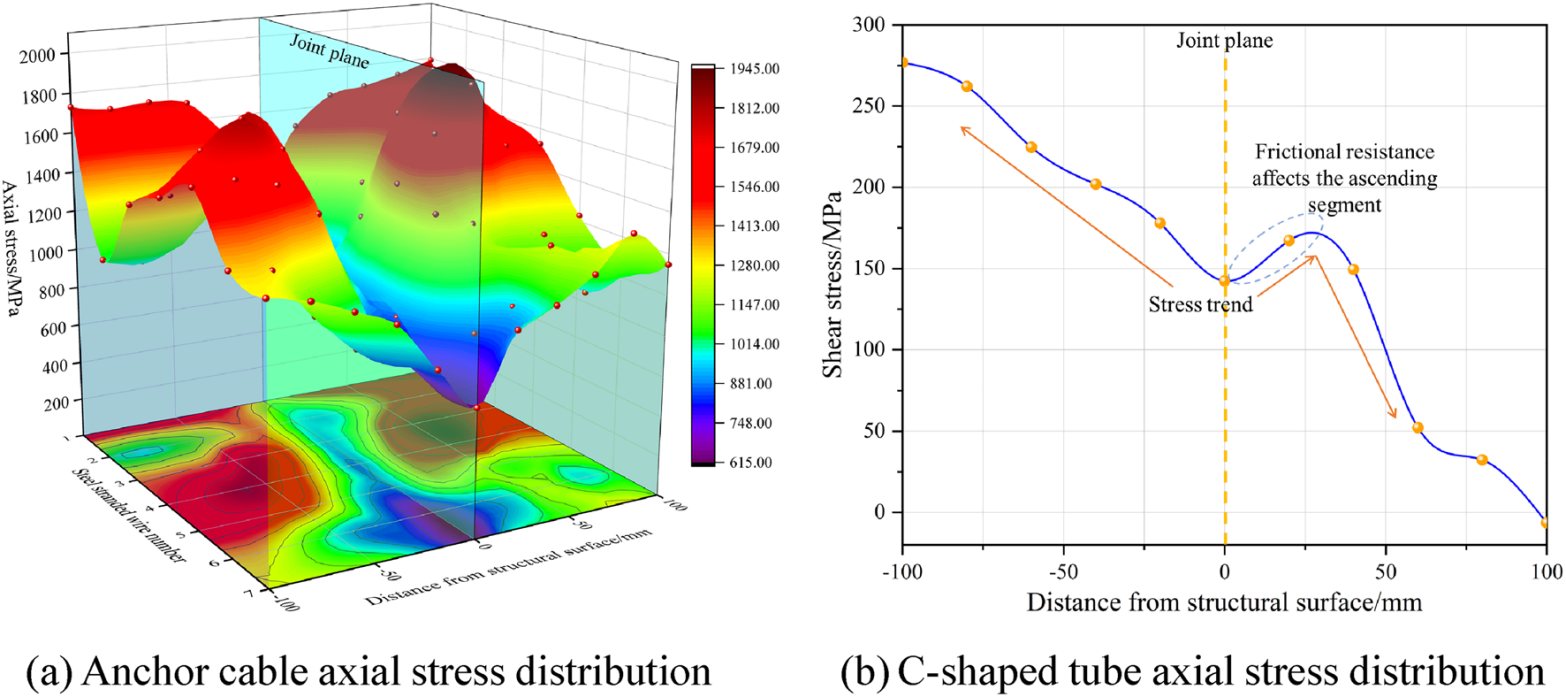
Distribution of axial stresses in ACC structural components.

For the distribution of axial stresses shown in Fig. 18, the strand exhibits the opposite distribution of shear stresses. With increasing distance from the structural surface, there is a notable increase in axial stress; furthermore, the stress distribution pattern exhibits symmetry relative to the structural surface. The axial stress observed in the C-shaped tube exhibits a declining trend within the range of − 100 to +100. There is a pattern of symmetrical distribution within a range of ± 20 mm. The observed rise within the 0–20 mm range is attributable to a significant increase in frictional forces over the non-closed section, engendered by the augmented contact area between the deformed closure of the C-shaped tube, the concrete block, and the internal anchoring cables, due to shear forces.

## Investigation of ACC deformation mechanism after C-shaped tube closure

**Figure 19:**
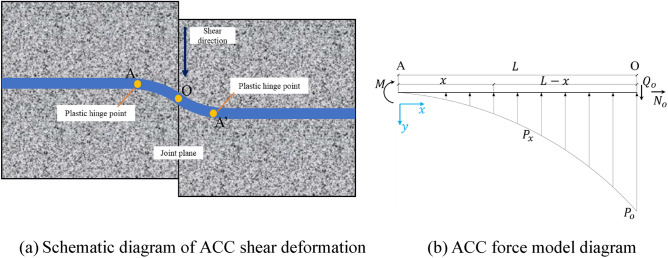
Schematic diagram of ACC deformation and force.

Once the C-tube closes, the internal anchor cables are entirely encapsulated within the ACC structure, representing a unified structure that restrains the surrounding rock's deformation. Research indicates that during shearing, as shear displacement increases, both anchor rods and cables develop symmetric plastic hinges on either side of the structural surface, as illustrated in Fig. 19a A-A′^24^; the position of these plastic hinges from the support elements is contingent upon the rock mass strength. With increasing rock mass strength, positions A and A′ progressively move towards point O on the structural interface until tension-shear failure falls on the support elements at that locus. Ferrero^25^ concluded that during the creation of plastic hinges in shear tests for anchor rods and cables, the shear force peaks at point O, where the bending moment is nil; conversely, at points A and A', the shear force is null and the bending moment is at its zenith.

The deformation and failure modes of the ACC structure parallel those of the anchor rod and cable system, with forces at the structural interface being anti-symmetrical, permitting dissection along the nodal plane for independent force examination. In this paper, the AO segment is modeled as a straight line to investigate the deformation mechanism of the ACC structure. Figure 19b shows the force on the ACC between the joint plane and the plastic hinge. Where $P_{(x)}$ is the rock reaction force, which is parabolically distributed in the AO section, $Q_{o}$ is the shear force of the ACC structure at O, and $N_{o}$ is the axial force of the anchor cable.

Establish a right-angled coordinate system with point A as the origin to obtain the expression for the rock reaction force in the AO section of the rock mass:

9 $$P(x) = \frac{{P_{o} }}{{L^{2} }}x^{2} ,$$

To derive the expression for the shear force $Q(x)$ in any cross-section of the ACC structure, one must formulate the equilibrium equations for the Y-axis and integrate Eq. (9):

10 $$Q(x) = Q_{o} - \int_{x}^{L} P (x)dx = Q_{o} - \frac{{P_{o} L}}{3} + \frac{{P_{o} x^{3} }}{{3L^{{^{2} }} }},$$

By integrating the shear force $Q(x)$, one obtains an expression for the bending moment $M(x)$ at any cross-section:

11 $$M(x) = \int_{x}^{L} Q \left( x \right)dx = Q_{o} (L - x) - \frac{{P_{o} L^{2} }}{4} + \frac{{P_{o} Lx}}{3} - \frac{{P_{o} x^{4} }}{{12L^{2} }},$$

Owing to the properties of the ACC structure, it can be equated to a flexible straight beam structure with uniform bending stiffness $E_{A} I_{A}$. The corresponding differential equation for its deflection curve may thus be represented as:

12 $$E_{A} I_{A} \frac{{d^{2} \omega }}{{dx^{2} }} = M(x).$$

The corner $\theta$ expression can then be expressed as:

13 $$\theta (x) = \frac{d\omega }{{dx}} = \frac{1}{{E_{A} I_{A} }}\left( {Q_{o} Lx - \frac{{Q_{o} x^{2} }}{2} - \frac{{P_{o} L^{2} x}}{4} - \frac{{P_{o} x^{5} }}{{60L^{2} }} + \frac{{P_{o} Lx^{2} }}{6} + C} \right)$$

The deflection $\omega$ is expressed as:

14 $$\omega (x) = \frac{1}{{E_{A} I_{A} }}\left( {\frac{{Q_{o} Lx^{2} }}{2} - \frac{{Q_{o} x^{3} }}{6} - \frac{{P_{o} L^{2} x^{2} }}{8} - \frac{{P_{o} x^{6} }}{{360L^{2} }} + \frac{{P_{o} Lx^{3} }}{18} + Cx + D} \right),$$

where $C$, $D$ are constants to be determined, from the boundary conditions: $x = 0$, $\theta (0) = 0$, $\omega (0) = 0$, can be obtained $C = D = 0$.

Therefore, the corner $\theta_{o}$ and deflection $\omega_{o}$ of the ACC structure at the structural face can be obtained from Eqs. (13) and (14):

15 $$\left\{ {\begin{array}{*{20}l} {\theta_{o} = \frac{1}{{E_{A} I_{A} }}\left( {\frac{1}{2}Q_{o} L^{2} - \frac{1}{10}P_{o} L^{3} } \right)} \hfill \\ {\omega_{o} = \frac{1}{{E_{A} I_{A} }}\left( {\frac{1}{3}Q_{o} L^{3} - \frac{13}{{180}}P_{o} L^{4} } \right)} \hfill \\ \end{array} } \right..$$

For the yield condition of ACC structures, the established judgment criteria are applicable, yet the results are imprecise and associated with a certain error. Consequently, an additional yield criterion needs to be introduced. Force analysis of the ACC structure reveals that its damage is primarily characterized by a combination of tensile and shear damage, and the stress state conforms to the Von-Mises criterion, that is:

16 $$\sigma_{y} = \sqrt {\sigma^{2} + 3\tau^{2} } = \sqrt {(\frac{{N_{o} }}{{A_{c} }})^{2} + 3(\frac{{Q_{o} }}{{A_{A} }})^{2} } .$$

Here, $\sigma_{y}$ represents the ACC yield stress, $\sigma$ corresponds to the axial stress, $\tau$ denotes the tangential stress, $A_{c}$ is the cross-sectional area of the anchor cable, and $A_{A}$ is the cross-sectional area of the ACC structure. In order to establish the connection between $N_{o}$ and $Q_{0}$ and the rock reaction force $P_{o}$, it is essential to analyze the relationship of energy within the ACC structures during shear deformation. When the shear deformation occurs in the ACC structure, the tensile deformation also occurs, and when the stress does not exceed the proportional limit, Hooke's law is satisfied. Therefore, it can be postulated that the axial deformation of the ACC structure increases linearly, and this axial deformation at the joint plane is denoted as $u_{o}$. The axial displacement $u(x)$ of ACC structure can be expressed by the following formula:

17 $$u(x) = \frac{{u_{o} }}{L}x.$$

Then the expression of variable situation energy is:

18 $$U = \frac{1}{2}\int_{0}^{L} {\left[ {E_{A} A_{A} (\frac{du}{{dx}})^{2} + E_{A} I_{A} (\frac{{d^{2} \omega }}{{dx^{2} }})^{2} } \right]} dx.$$

The work done by an external force is expressed as follows:

19 $$W = N_{o} u_{o} + Q_{o} \omega_{o} - \int_{0}^{L} P (x)\omega (x)dx.$$

The total potential energy $\Pi$ of the system can be represented as the sum of variable situation energy $U$ and work done by external force $W$:

20 $$\Pi = U - W,$$

Integrating Eqs. (9), (14), (17), (18), (19) and (20) derives an expression for the system's total potential energy:

21 $$\Pi = \frac{{E_{A} A_{A} u_{o}^{2} }}{2L} - N_{o} u_{o} + \frac{{L^{3} \left( {306P_{o} Q_{o} L - 540Q_{o}^{2} - P_{o}^{2} L^{2} \left( {25 + L^{2} } \right)} \right)}}{{3240E_{A} I_{A} }}.$$

According to the principle of minimum potential energy, the following can be obtained:

22 $$\left\{ {\begin{array}{*{20}l} {\frac{\partial \Pi }{{\partial u_{o} }} = \frac{{E_{A} A_{A} u_{o} }}{L} - N_{o} = 0} \hfill \\ {\frac{\partial \Pi }{{\partial Q_{o} }} = \frac{{17P_{o} L^{4} }}{{180E_{A} I_{A} }} - \frac{{Q_{o} L^{3} }}{{3E_{A} I_{A} }} = 0} \hfill \\ \end{array} } \right..$$

Simplification can be obtained:

23 $$\left\{ {\begin{array}{*{20}l} {N_{o} = \frac{{E_{A} A_{A} u_{o} }}{L}} \hfill \\ {Q_{o} = \frac{{17P_{o} L}}{60}} \hfill \\ \end{array} } \right..$$

During the shearing process, it is anticipated that the deformation of the ACC structure will adhere to a specific coordination relationship. To simplify the computational process, it is postulated that within the ACC structure’s deformation progression, point $O_{1}$ corresponds to point $O$ under pure flexural deformation, point $O_{2}$ aligns with point $O$ along the vertical trajectory of the flexural curve, and point $O_{3}$ coincides with point $O$ following shear deformation. $O_{1} O_{2}$ represents the axial deformation length within the ACC structure, whereas $O_{2} O_{3}$ quantifies the slip distance ensuing from the disconnection between the anchor cable and its corresponding locking mechanism.

**Figure 20:**
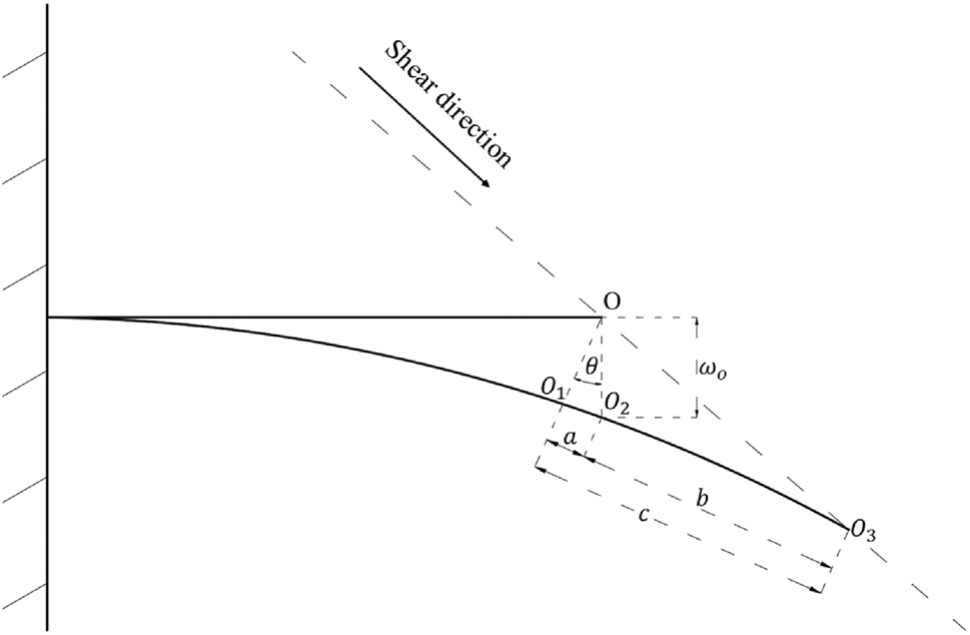
Schematic diagram of ACC deformation coordination relationship.

Based on the relationship depicted in Fig. 20, it can be derived that:

24 $$a = \omega_{o} \sin \theta = u_{o} .$$

When the structure undergoes a minor deformation, it may be approximated that $\sin \theta = \theta$. Therefore, by combining the formulas (15), (16), (23) and (24), the stress state relation of ACC structure at yield can be obtained:

25 $$\sigma_{y} = \sqrt {(\frac{{P_{o}^{2} L^{6} }}{{1080E_{A} I_{A}^{2} }})^{2} + 3 \cdot (\frac{{17P_{o} L}}{{60A_{A} }})^{2} } .$$

Considering that the ACC structure can be viewed as a unified system once the C-shaped tube is closed, its modulus of elasticity $E_{A}$ and the sectional moment of inertia $I_{A}$ are linked by approximately the following relationship:

26 $$\left\{ {\begin{array}{*{20}l} {E_{A} = \frac{{E_{B} A_{B} + E_{C} A_{C} }}{{A_{B} + A_{C} }}} \hfill \\ {I_{A} = \frac{{\pi \left( {2\delta + 2R_{B} } \right)^{4} }}{64} = \frac{{\pi \left( {\delta + R_{B} } \right)^{4} }}{4}} \hfill \\ \end{array} } \right..$$

Where $E_{B}$ is the elastic modulus of the anchor cable, $A_{B}$ is the cross-sectional area of the anchor cable, $E_{C}$ is the elastic modulus of the C-shaped tube, $A_{C}$ is the cross-sectional area of the C-shaped tube, $\delta$ is the wall thickness of the C-shaped tube, and $R_{B}$ is the radius of the anchor cable.

**Figure 21:**
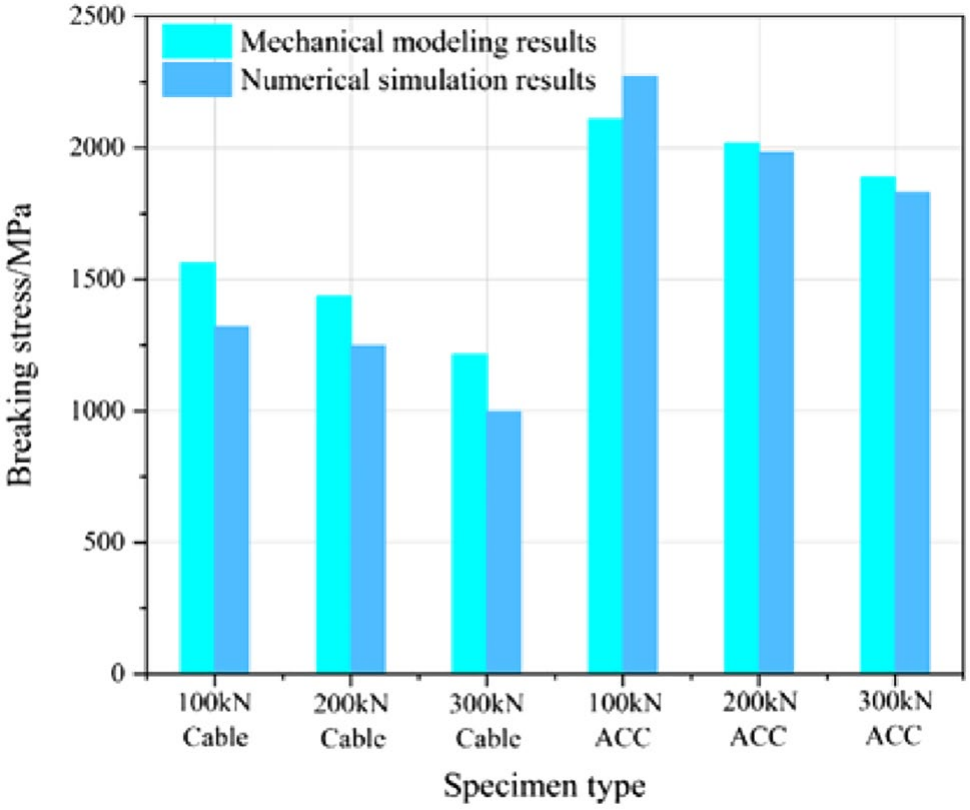
Comparison of numerical simulation and mechanical modeling results.

The relationship curve between the rock reaction force $P_{o}$ and the yield stress $\sigma_{y}$ of the ACC structure following the closure of the C-shaped tube is depicted in Fig. 20. The accuracy of the resultant theoretical model was confirmed by substituting the rock reaction force $P_{o}$ into the ACC structural yield state relationship equation, also validated for common anchor cable structures and the comparative results are depicted in Fig. 21.

Figure 21 demonstrates that the disparity between the mechanical model calculations and the numerical simulation measurements of the fracture stress of the ACC structure is minimal, with an average variance of around 6.0%. Nevertheless, when using this mechanical model to compute the fracture stress of conventional anchor cable constructions, the outcomes exhibit notable disparities compared to the values derived from numerical simulation, with an average discrepancy of around 15.5%. Hence, this mechanical model exhibits limited suitability for conventional anchor cable systems and is more appropriate for specialized constructions such as ACC.

## Conclusion

In this paper, a comparative study of free-section double-shear tests on anchor cable structures and ACC structures was carried out by applying an indoor double-shear testing machine. This paper details using an indoor double-shear testing apparatus to perform comparative analyses of free-section bi-directional shear tests on anchor cable structures and ACC structure. Further, a detailed simulation analysis of the double- shear tests on ACC configurations was executed utilizing the ABAQUS finite element analysis software, which aimed to elucidate the deformation mechanisms of ACC structures under shear-induced yielding. The main conclusions of the study are as follows:

1. Owing to the incorporation of a C-shaped tube within the ACC structure, there is a reduction in stress concentration during the shear deformation process. At the same time, improve the stress state of internal anchor cable structure. As a result, the failure pattern of the tension-shear combination has shifted, with tensile breaking becoming the predominant form of rupture.
2. The numerical model of the twin shear test for ACC structures is capable of accurately reflecting the deformation development stages under different prestresses. When monitoring and analyzing the internal stress distribution is infeasible, the numerical model can distinctly delineate the axial and shear stress development and distribution within each component of the ACC structure, as well as the resultant failure pattern of the rock mass.
3. Based on the principles of the statically determinate beam theory, in conjunction with the minimum potential energy principle and the mises yield criterion, the shear deformation model of the ACC structure can succinctly articulate the overall stress variation within the ACC structure and elucidate the relationship between internal stress and the rock mass reaction during the deformation process of the ACC structure.

## Data Availability

The datasets used and analyzed during the current study are available from the corresponding author on reasonable request.

## References

[1] Xuezhen W, Jiang Y, Bo L (2018). Influence of joint roughness on the shear behaviour of fully encapsulated rock bolt. Rock Mech. Rock Eng..

[2] Xuezhen W, Yujing J, Gong B, Zhenchang G, Tao D (2019). Shear performance of rock joint reinforced by fully encapsulated rock bolt under cyclic loading condition. Rock Mech. Rock Eng..

[3] JinHua W (2012). Analysis on mechanism and effect of rock bolts and cables in gate road with coal seam as roof. J. China Coal Soc..

[4] Kang H, Yang J, Gao F, Li J (2020). Experimental study on the mechanical behavior of rock bolts subjected to complex static and dynamic loads. Rock Mech. Rock Eng..

[5] Aziz N, Mirzaghorbanali A, Nemcik J, Heemann K, Mayer S (2015). Shear strength properties of plain and spirally profiled cable bolts. Can. Geotech. J..

[6] Aziz, N., Mirza, A., Nemick, J., Xuwei, L. & Rasekh, H. Load transfer characteristics of plain and spiral cable bolts tested in new non rotating pull testing apparatus. In Coal operators’ conference, University of Wollongong, p32-39 (2016).

[7] Aziz N (2018). An experimental study on the shear performance of fully encapsulated cable bolts in single shear test. Rock Mech. Rock Eng..

[8] Craig, P., Aziz, N. Shear testing of 28 mm hollow strand "TG" cable bolt. In Coal Operators Conference, 171-179 (2010).

[9] Li X, Aziz N, Mirzaghorbanali A, Nemcik J (2016). Behavior of fiber glass bolts, rock bolts and cable bolts in shear. Rock Mech. Rock Eng..

[10] Li X, Nemcik J, Mirzaghorbanali A, Aziz N, Rasekh H (2015). Analytical model of shear behaviour of a fully grouted cable bolt subjected to shearing. Int. J. Rock Mech. Min..

[11] Rasekh H (2017). Double shear testing of cable bolts with no concrete face contacts. Procedia Eng..

[12] Li L, Hagan PC, Saydam S, Hebblewhite B, Li Y (2016). Parametric study of rockbolt shear behaviour by double shear test. Rock Mech. Rock Eng..

[13] Sun B (2022). Numerical implementation of rock bolts with yield and fracture behaviour under tensile-shear load. Eng. Fail. Anal..

[14] Nie W, Zhao ZY, Ning YJ, Guo W (2014). Numerical studies on rockbolts mechanism using 2D discontinuous deformation analysis. Tunn. Undergr. Space Technol..

[15] Aziz N (2022). Angle shear testing of 15.2 mm seven wire cable bolt. Rock Mech. Rock Eng..

[16] Tahmasebinia F, Zhang C, Canbulat I, Vardar O, Saydam S (2018). Numerical and analytical simulation of the structural behaviour of fully grouted cable bolts under impulsive loading. Int. J. Min. Sci. Technol..

[17] Tahmasebinia F (2021). A new concept to design combined support under dynamic loading using numerical modelling. Tunn. Undergr. Space Technol..

[18] Shan R (2023). Study on asymmetric support of anchor cable with C-shaped tube in inclined coal seam roadway. Appl. Sci..

[19] Shan R (2022). Behavior of anchor cable bolts with c-shaped tube and cable bolts in shear test. AIP Adv..

[20] Shan R (2022). Experimental study on the shear mechanical properties of anchor cable with C-shaped tube. Sustainability.

[21] Shan R (2022). Research on the anchor cable combined with the c-shaped tube and the mechanical properties. Rock Soil Mech..

[22] Shan R (2023). Research on the new technology of anchor cable with C-shaped tube support and its application in deep large deformation roadway. J. Min. Sci. Technol..

[23] Wang K, Zhao Y, Hu Z, Nie Y (2023). Shear test of pre-stressed anchor block and fracture mechanism analysis of anchor cable. Rock Mech. Rock Eng..

[24] Pellet F, Egger P (1996). Analytical model for the mechanical behaviour of bolted rock joints subjected to shearing. Rock Mech. Rock Eng..

[25] Ferrero AM (1995). The shear-strength of reinforced rock joints. Int. J. Rock Mech. Min..

